# Development of certified reference materials for the determination of cadmium and acrylamide in cocoa

**DOI:** 10.1007/s00216-020-02719-0

**Published:** 2020-06-02

**Authors:** Sebastian Recknagel, Matthias Koch, Robert Köppen, Sabine Buttler, Sibylle Penk, Tatjana Mauch, Thomas Sommerfeld, Angelika Witt

**Affiliations:** Bundesanstalt für Materialforschung und -prüfung (BAM), Richard-Willstätter-Straße 11, 12489 Berlin, Germany

**Keywords:** Certified reference material, Quality assurance, Cocoa, Cadmium, Acrylamide, Food analysis

## Abstract

Since 1 January 2019 a maximum content of 0.6 mg kg^−1^ cadmium (Cd) in cocoa powder sold to the final consumer or as an ingredient in sweetened cocoa powder sold to the final consumer (drinking chocolate) is set by the Commission Regulation (EU) No. 488/2014. Monitoring compliance with the specified limit value requires analytical measuring methods and reference materials for quality control. However, suitable certified reference materials intended for quality assurance and quality control purposes are still lacking. Therefore, three cocoa reference materials (ERM®-BD513, ERM®-514 and ERM®-515) were developed according to the requirements of ISO 17034 and the recommendations of ISO Guide 35. The whole process of reference material development, including material preparation, assessment of homogeneity and stability, characterisation and value assignment is presented. The assignment of the certified mass fractions was based upon an interlaboratory comparison study involving 19 expert laboratories for Cd and 12 laboratories for acrylamide. The certified mass fractions and expanded uncertainties (*k* = 2) of the reference materials were (0.181 ± 0.009) mg kg^−1^ Cd (ERM®-BD513), (0.541 ± 0.024) mg kg^−1^ Cd (ERM®-BD514) and (0.690 ± 0.029) mg kg^−1^ Cd (ERM®-BD515). Acrylamide contents are given for information.

## Introduction

Food safety and quality are two of the most important factors determining consumer acceptance of a product. Proof of authenticity, fraud detection and the detection of residues and harmful substances, e.g. heavy metals like cadmium and lead are therefore the focus of general interest.

A biological function of cadmium in animals and humans is not known, but cadmium can mimic other divalent metals that are essential for various biological functions, e.g. calcium in bone [1]. Due to the occurrence of the Itai-Itai disease in Japan in the mid-1950s of the twentieth century, the toxic effects of cadmium for humans are well known. Especially the kidneys and bones are affected by cadmium intake [1].

Commissioned by the European Commission, the Panel on Contaminants in the Food Chain (CONTAM Panel) of the European Food Safety Authority (EFSA) issued a new opinion on cadmium (Cd) in food in 2009 [2]. Since chocolate and cocoa products are one of the food groups that contribute most to dietary exposure to Cd, and since chocolate as such and other sweetened cocoa products used in cocoa drinks are often consumed by children and adolescents, the European Commission has decided to set a limit for Cd in cocoa and chocolate. The EU Commission Regulation (EU) No. 488/2014 of 12 May 2014 sets a maximum content of 0.6 mg Cd kg^−1^ in cocoa powder sold to the final consumer or as an ingredient in sweetened cocoa powder sold to the final consumer (drinking chocolate) from 1 January 2019.

For the implementation of the food and feed law there is a great need for the development and harmonisation of analytical methods. Whenever possible, official food control laboratories must use validated methods. Therefore, methods of analysis must be subject to validation procedures in order to provide accurate, repeatable and reproducible results within and between laboratories. The purpose of method validation is to verify the performance of a method. Food and feed reference materials and in particular certified reference materials (CRMs) are a very useful tool for checking the accuracy of analytical measurements. They can be used to estimate the measurement uncertainty, to assess the metrological traceability of analytical results or to calibrate analytical equipment.

In view of the Cd limit set by the EU Commission, it is necessary to analyse cocoa and cocoa products for their Cd content and Bundesanstalt für Materialforschung und -prüfung (BAM) has therefore decided to produce a series of cocoa CRMs with increasing cadmium contents around the official EU maximum levels. Also in view of the new EU regulation, the JRC (Joint Research Centre) prepared a chocolate CRM (ERM®-BD512) certified for its mass fractions of Cd and Cu, Mn and Pb [3]. Similar reference materials are not available although there are a couple of food CRMs with certified Cd mass fractions, e.g. rice flour [4, 5], milk powder and seafood [6].

It was decided to characterise the cocoa powder reference materials also with regard to their mass contents of acrylamide, as acrylamide is also a contaminant of interest in food. Since Swedish scientists reported the presence of acrylamide in many baked or fried foods in April 2002, worldwide monitoring of this substance in food has started [7, 8] and there are only a few CRMs certified for acrylamide [9, 10].

The three candidate reference materials ERM®-BD513, ERM®-BD514 and ERM®-BD515 to be certified were produced within the ERM® initiative (European Reference Materials) for the purpose of quality assurance and quality control for the determination of cadmium and acrylamide in food. The materials have been prepared from cocoa powder from commercial sources intended for human consumption.

Sixteen external and three internal laboratories were selected based on documented experience and proficiency and invited to participate in the certification study of the candidate materials prepared at BAM. Certification was carried out on the basis of ISO 17034 [11] and the relevant ISO Guides [12, 13].

### Material preparation

In some regions of cocoa-producing countries, the cadmium content of the soil can be naturally high. It is therefore to be expected that different types of cocoa are contaminated with Cd in different ways depending on their place of origin. A total of nine different commercially available cocoa powders (K1–K9) were purchased from food retailers and subsequently analysed for their Cd content. Three of them with Cd contents around the Cd limit were chosen as candidate materials for the three reference materials. In total 4.65 kg of K8 (ERM®-BD513), 7.43 kg of K9 (ERM®-BD514) and 8.25 kg of K7 (ERM®-BD515) were taken as starting material. Approximately 90% of the powder had a particle size between 50 and 100 μm.

The three selected batches were homogenised in a drum-hoop mixer for 24 h each using a stainless-steel drum. After homogenisation, the candidate reference materials were filled in aluminised plastic bags using a 10-division sample divider PT 100 (Retsch, Haan, Germany). Each plastic bag was filled with at least 8.3 g of cocoa powder. In total, 500 bags of ERM®-BD513, 760 bags of ERM®-BD514 and 800 bags of ERM®-BD515 were obtained. Immediately after filling the plastic bags were stored at − 21 °C in a freezer.

For secondary matrix , 15 bags from each batch were selected and analysed by coulometric Karl Fischer titration. Table 1 shows the results of the moisture determination.

**Table 1:** Moisture *M* of ERM®-BD513, ERM®-BD514 and ERM®-BD515 (*n* = 45), determined by coulometric Karl Fischer Titration (*s*: standard deviation)

ERM®	*M* (g/100 g)	*s* (g/100 g)
BD513	5.04	0.08
BD514	3.70	0.19
BD515	3.58	0.08

### Analytical methods

Characterisation of potential candidate material as well as homogeneity and stability assessment on the finally chosen candidate materials were carried out using the following analytical procedures:

#### Moisture determination

Karl Fischer titration for the determination of the moisture of the cocoa samples was performed using the oven drying method with an oven temperature of 120 °C. Dried nitrogen (80 mL min^−1^) was used to purge the heated transfer line between oven and coulometric titration vessel. A total of 100–120 mg of sample was weighed for the analysis. The whole determination was carried out using an 851 Titrando system with automatic sampler (Metrohm 874 Oven Sample Processor), stirrer (Metrohm 801 Stirrer) and software tiamo™ 2.5 for method parameter, analysis control and data evaluation (all Metrohm, Herisau, Switzerland). No calibration was necessary since coulometry is a primary method. Quality control check was done by analysing certified water standards HYDRANAL-Water Standard KF-Oven, (5.04% ± 0.03%, Honeywell Fluka, Schwerte, Germany).

#### Cadmium

Based on the procedures described in EN 13805 [14] and EN 15763 [15] 0.5 g of cocoa powder were digested with 4 mL of HNO_3_ (65%, cleaned by subboiling distillation, Merck, Darmstadt, Germany) and 1 mL of H_2_O_2_ (p.a., Merck) in an ultraCLAVE digestion unit (MLS, Leutkirch, Germany). The test sample was used as received from the plastic bag, i.e. without any pretreatment like drying. Cd determination in the clear sample solutions received from microwave digestion was carried out with inductively coupled plasma mass spectrometry (ICP-MS) using a quadrupole ICP-mass spectrometer 7500cs (Agilent Technologies, Santa Clara, CA, USA) equipped with a micro flow nebuliser. Calibration was carried out using five acidified standard solutions prepared from a 1000 mg L^−1^ Cadmium ICP standard (Merck Certipur directly traceable to primary NIST SRM® 3108, Merck). All measurements were performed using the ^111^Cd isotope, ^103^Rh as internal standard and He as reagent gas in the reaction cell (plasma power: 1500 W).

#### Acrylamide

50 mg of the powdered cocoa sample were placed in a 2 mL snaplock microtube and covered with 1.5 mL water containing the internal standard (^13^C_3_-acrylamide, ~ 20 ng g^−1^, CIL Cambridge Isotope Laboratories, Tewksbury, MA, US) and ninhydrin (10 mg g^−1^, > 95%, Sigma-Aldrich, St. Louis, Missouri, USA). Samples were extracted for 2 h at 1100 rpm in an MHR 13 thermomixer (HLC BioTech, Bovenden, Germany) at 85 °C, which is well below the formation temperature of acrylamide [16], and subsequently centrifuged for 10 min by means of a micro-centrifuge (Eppendorf Minispin® plus, Eppendorf, Hamburg, Germany) at 14,100×*g*. The aqueous phase was transferred to a second snaplock microtube by using a syringe fitted with a needle, avoiding the transfer of the upper lipid phase. Afterwards, an aliquot (1.2 mL) was transferred to another snaplock microtube. Visual protein precipitation was achieved by addition of 150 μL of a 0.68 M potassium hexacyanoferrate(II) trihydrate solution and 150 μL of a 2 M zinc sulfate heptahydrate solution (both > 98.5%, Sigma-Aldrich, St. Louis, Missouri, USA). After short shaking, the mixture was centrifuged for 2 min at 14,100×*g* and a 0.8 mL aliquot was transferred to a snaplock microtube. This aliquot was extracted three times with 1 mL of ethyl acetate (Chemosolute®, Th. Geyer, Renningen, Germany) followed by shaking and a centrifugation step (0.5 min, 14,100×*g*). The organic supernatants were combined in an 8-mL vial and 1 mL of water was added to serve as a keeper during solvent evaporation and prevent loss of the analyte by keeping it dissolved. Subsequently, the ethyl acetate layer was evaporated under a gentle stream of nitrogen at 30 °C. The resulting aqueous solution was loaded onto a 100 mg Isolute® Multimode SPE cartridge, preconditioned with first 1 mL of acetonitrile (Chemosolute®, Th. Geyer) and then 3 mL of water. The aqueous solution was eluted and collected in a 4-ml vial. One milliliter of water was loaded onto the cartridge, eluted, and collected in the same 4-mL vial of the previous fraction. Afterwards, the eluate was passed through a 0.2 μm membrane filter and collected in a 1.5-mL vial for LC-ESI-MS/MS analysis. The chromatographic separation was performed with an Agilent® 1260 HPLC (Agilent Technologies, Inc., Santa Clara, CA, USA) with a Waters® Atlantis TM T3, 150 × 2.1 mm, 3-μm column (Waters Corporation, Milford, MA 01757 USA) using a gradient of H_2_O + 0.1% formic acid as mobile phase A and methanol (both Chemosolute®, Th. Geyer) as mobile phase B. Mass spectrometric measurements were performed using a SCIEX Triple Quad API 4000 QTRAP® (AB Sciex LLC, Framingham, MA, USA). Calibration was performed in the range of 2–12 ng acrylamide (Ultra Scientific, North Kingstown, RI, USA) with 7 calibration points (regression coefficient *r* = 0.996) using a certified acrylamide standard (Ultra Scientific).

### Assessment of homogeneity

The homogeneity study assesses the distribution of the components to be certified in all the units bottled. That means that uncertainty contribution resulting from possible heterogeneity (between-unit inhomogeneity) has to be quantified in terms of contents for Cd and acrylamide, among randomly chosen bags. For that purpose, 15 units were randomly selected from each of the three batches and analysed three times each under repeatability conditions in one run using one calibration according to the analytical method described above.

The estimates of analyte-specific inhomogeneity contributions *u*_bb_ to be included into the total uncertainty budgets were calculated according to ISO Guide 35 [12] using Eq. (1):

1 $$ {u}_{\mathrm{bb}}=\sqrt{\frac{MS\mathrm{among}\kern0.5em - MS\mathrm{within}}{n}} $$

- where

*MS*_among_: mean of squared deviations between bottles (from 1-way ANOVA)
*MS*_within_: mean of squared deviations within bottles (from 1-way ANOVA)
*n*: number of replicate subsamples per bottle

Equation (1) does not apply if *MS*_within_ is larger than *MS*_among_. In this case, the uncertainty contribution is set to zero (Cd in ERM®-BD513 and acrylamide in ERM®-BD513 and ERM®-BD515).

### Assessment of stability

From experience with other food materials, a temperature-driven deterioration especially of the acrylamide content was to be expected for cocoa [9, 10]. Selected units of the candidate materials were submitted to accelerated ageing at temperatures between − 21 and 60 °C over periods of 1 to 4 weeks (short-term study) and 1 to 12 months (long-term study) as shown in Table 2 to perform so-called isochronous stability studies [17]. Some units were stored at − 80 °C on the assumption that no degradation of acrylamide or loss of Cd or matrix occurs at this temperature. Based on this assumption, which has been confirmed by investigations of potato chips by Kim et al. [10], the units stored at − 80 °C were used as reference for the stability measurements.

**Table 2:** Accelerated ageing of exposed samples

	Temperature (°C)				
	− 80	− 21	23	40	60
Time (weeks)	Short-term stability study				
1	X	X	X	X	X
2	X	X	X	X	X
3	X	X	X	X	X
4	X	X	X	X	X
Time (months)	Long-term stability study				
3	X	X	X	X	–
6	X	X	X	X	–
9	X	X	X	X	–
12	X	X	X	X	–
48	^1^)	^1^)	–	–	–

^1^)Post-certification monitoring (due)

After the respective periods of time individual units were stored at − 80 °C. At the end of the 4-week-period chosen for short-term stability monitoring, all sample units were analysed for acrylamide as well as for Cd using the methods described above under repeatability conditions. The same procedure was applied to the samples stored for the study of long-term stability which were analysed after the 1-year period. This investigation was only performed for Cd since the results of the short-term stability investigation for acrylamide showed a relatively strong deterioration within the short-term stability period. Further investigations are planned as part of post-certification monitoring.

The data processing and evaluation of results was carried out in accordance with the approach described by Bremser et al. [18] under the assumption of an *Arrhenius* model for the dependence of the effective reaction rate *k*_eff_(T) on temperature. This kinetic approach was used to estimate the shelf life of the material especially for acrylamide at different storage temperatures.

### Characterisation and value assignment

The assignment of the certified Cd mass fractions of the candidate reference materials as well as the informative values for acrylamide contents was based upon an interlaboratory comparison with a total of 19 laboratories for Cd and 12 laboratories for acrylamide. Each participating laboratory received two bags of each candidate material and for measurement control purposes quality control samples for Cd and/or for acrylamide for direct analysis. The laboratories were asked to analyse four subsamples, two subsamples from each bag. They were free to choose any suitable analytical method. In addition, the participants received control solutions with known contents of Cd and acrylamide which they should analyse together with the cocoa samples to check their recovery rates.

Most of them determined Cd using ICP-MS or graphite furnace atomic absorption spectrometry (GFAAS) after decomposition of the cocoa powder with HNO_3_/H_2_O_2_ or HNO_3_/HCl in a microwave oven. One laboratory used Isotope dilution mass spectrometry after decomposition of the cocoa powder with HNO_3_/H_2_O_2_ in a high-pressure asher (Table 3).

**Table 3:** Analytical methods used for Cd determination

Laboratory	Sample intake (g)	Sample pretreatment	Analytical method	Calibration
L01	0.5	Dissolution with HNO_3_/H_2_O_2_; microwave	ICP-MS	CMS* (Inorganic ventures**)
L03	0.5	Dissolution with HNO_3_/H_2_O_2_; microwave	GFAAS	CMS (GUM**)
L04	0.5	Dissolution with HNO_3_/H_2_O_2_; microwave	ICP-MS	CMS (Ultra Scientific**)
L05	1	Dissolution with HNO_3_	ICP-MS	Pure metal (Sigma-Aldrich**)
L07	0.4	Dissolution with HNO_3_ under pressure acc. to DIN EN 13805:2014:2010	ICP-MS	CMS (Labkings**)
L09	0.5	Dissolution with HNO_3_/HCl	ICP-MS	CMS (Merck**)
L10	0.5	Dissolution with HNO_3_/H_2_O_2_; microwave	ICP-MS	CMS (Romil**)
L11	0.35	Dissolution with HNO_3_/H_2_O_2_; microwave acc. to EN 1404:2003	GFAAS	CMS (Roth**)
L12	0.5	Pressure digestion with HNO_3_/H_2_O_2_	ICP-MS	CMS (Spex**)
L14	0.4	Dissolution with HNO_3_; microwave	ICP-MS	CMS (Merck**)
L15	0.5	Dissolution with HNO_3_/H_2_O_2_; microwave	ICP-MS	CMS (Merck**)
L16	0.5	Pressure digestion with HNO_3_ acc. to DIN 13805:2010	GFAAS	CMS (Merck**)
L17	0.5	Dissolution with HNO_3_/HCl/H_2_O_2_ microwave	GFAAS	CMS (Merck**)
L18	0.5	Dissolution with HNO_3_/HCl; microwave	ICP-MS	CMS (VWR**)
L19	0.3	Dissolution with HNO_3_/H_2_O_2_; microwave	ICP-MS	CMS (Sigma-Aldrich**)
L20	0.5	Dissolution with HNO_3_/H_2_O_2_; microwave	ICP-MS	CMS (NIST**)
L21	0.5	Dissolution with HNO_3_/H_2_O_2_, high-pressure microwave	ICP-MS	CMS (Merck**)
L22	0.5	Dissolution with HNO_3_/H_2_O_2_, high-pressure microwave	GFAAS	CMS (Merck**)
L23	0.5	Dissolution with HNO_3_/H_2_O_2_, high-pressure asher	ID-MS	Spike ^113^Cd

*CMS commercial monoelement solution

**Inorganic ventures, Christiansborg, Virginia, USA; GUM – Central Office of Measures, Warsaw, Poland; Ultra Scientific, North Kingstown, RI, USA; Sigma-Aldrich, St. Louis, Missouri, USA; LabKings, Hilversum, The Netherlands; Merck, Darmstadt, Germany; Romil., Cambridge, UK; Roth, Karlsruhe, Germany; Spex, Metuchen, NJ, USA; VWR, Radnor, Pennsylvania, USA; NIST, Gaithersburg, Maryland, USA

For acrylamide determination, most of the participating laboratories used a procedure in accordance with EN 16618 [19] analysing acrylamide with liquid chromatography/mass spectrometry (LC-MS/MS) with deuterated acrylamide as internal standard (Table 4).

**Table 4:** Analytical procedures for acrylamide determination

Lab-No.	Sample intake (g)	Sample preparation	Analytical method	Calibration
L02	0.2	Extraction with methanol/water (60/40), centrifugation, filtration	LC-MS/MS, no internal standard (IS)	AA* 98% (CIL**)
L05	2	Extraction with water, centrifugation, filtration, SPE clean-up	LC-MS/MS, d_5_-acrylamide as IS	AA (Sigma-Aldrich**)
L06	2	according to EN 16618 (procedure for coffee)	LC-MS/MS, d_3_-acrylamide as IS	AA 98% Tracecert (Sigma-Aldrich**)
L07	1	extraction with water/methanol, centrifugation	LC-MS/MS, d_3_-acrylamide as IS	no information available
L08	1	according to EN 16618	LC-MS/MS, d_3_-acrylamide as IS	AA > 99% (Sigma-Aldrich**)
L10	2	Extraction with water, centrifugation, filtration, SPE clean-up, centrifugation with cut-off filter	LC-MS/MS, d_3_-acrylamide as IS	AA > 99% (Sigma-Aldrich**)
L11	1/2	according to EN 16618	LC-MS/MS, d_3_-acrylamide as IS	AA 99% (Merck**)
L12	2	Extraction with water, Carrez I and II clarification, SPE clean-up	LC-MS/MS, d_3_-acrylamide as IS	AA 99.3% (Ehrenstorfer**)
L16	2	Extraction with n-hexane + water, centrifugation, 2x SPE clean-up	LC-MS/MS, d_3_-acrylamide as IS	AA 99.9% (Sigma-Aldrich**)
L18	2	Extraction with water, SPE clean-up, L/L partitioning with ethylacetate	GC-MS, d_3_-acrylamide as IS	AA 99.9% (Alfa-Aesar**)
L20	2	Extraction with n-hexane + water, centrifugation, 2× SPE clean-up	LC-MS/MS, d_3_-acrylamide as IS	AA Tracecert (Fluka**)
L21	0.05	Extraction with water (+ ninhydrin), centrifugation, Carrez I and II clarification, centrifugation, L/L partitioning with ethylacetate, centrifugation, SPE clean-up	LC-MS/MS, ^13^C_3_-acrylamide as IS	AA (Ultra Scientific**)

*AA acrylamide

**CIL Cambridge Isotope Laboratories, Tewksbury, MA, USA; Sigma-Aldrich, St. Louis, Missouri, USA; Merck, Darmstadt, Germany; Ehrenstorfer (LGC), Augsburg, Germany; Alfa-Aesar, Haverhill, Massachusetts, USA; Honeywell Fluka, Schwerte, Germany; Ultra Scientific, North Kingstown, RI, USA

## Results and discussion

### Assessment of homogeneity

Based on a thorough homogenisation of the three cocoa batches before portioning, a satisfactory level of sample homogeneity was expected. This assumption was confirmed by the evaluation of the measurement data of the homogeneity tests. The results were evaluated using one-factorial ANOVA, the results are shown in Tables 5 and 6. Because the test statistic is lower than the critical value, no significant inhomogeneity of all three batches and both analytes was detected. According to ISO Guide 35, an inhomogeneity contribution to the total uncertainty has to be considered if *MS*_among_ is larger than *MS*_within_. This was the case for Cd in ERM®-BD514 and ERM®-BD515 and for acrylamide in ERM®-BD514. In the other cases, this uncertainty contribution was set to zero. It has to be pointed out that this is only valid for a minimum sample intake of 0.5 g in the case of Cd and 50 mg in the case of acrylamide. Therefore, the use of test portions less than the respective minimum sample intakes is prohibited in order to avoid possible inhomogeneities with smaller sample sizes.

**Table 5:** Analysis of variance (ANOVA) and estimates for uncertainty contribution according to ISO Guide 35 for cadmium

ERM®	MS_among_ (mg kg^−1^)	MS_within_ (mg kg^−1^)	Test statistic MS_among_/MS_within_	Critical value *F* (*f*1, *f*2; 5%)	*u*_bb_ (mg kg^−1^)
BD513	0.000004358	0.000007423	0.587029	2.037420	0
BD514	0.00005354	0.000029036	1.844061	2.037420	0.002858
BD515	0.00011566	0.000068098	1.698438	2.037420	0.003982

**Table 6:** Analysis of variance (ANOVA) and estimates for uncertainty contribution according to ISO Guide 35 for acrylamide

ERM®	*MS*_among_ (mg kg^−1^)	*MS*_within_ (mg kg^−1^)	Test statistic *MS*_among_*/MS*_within_	Critical value *F* (*f*1, *f*2; 5%)	*u*_bb_ (mg kg^−1^)
BD513	0.000023280	0.000024541	0.948499	2.037420	0
BD514	0.000086132	0.000070209	1.226789	2.037420	0.0023038
BD515	0.0000465	0.00004998	0.931231	2.037420	0

### Assessment of stability

The analytical data produced for each temperature and time scheme (data not shown) were evaluated. For cadmium, there was no evidence of any instability, neither in terms of short-term nor long-term stability. Therefore, no uncertainty contribution related to stability was included into the total uncertainty of the certified Cd mass fractions.

**Fig. 1:**
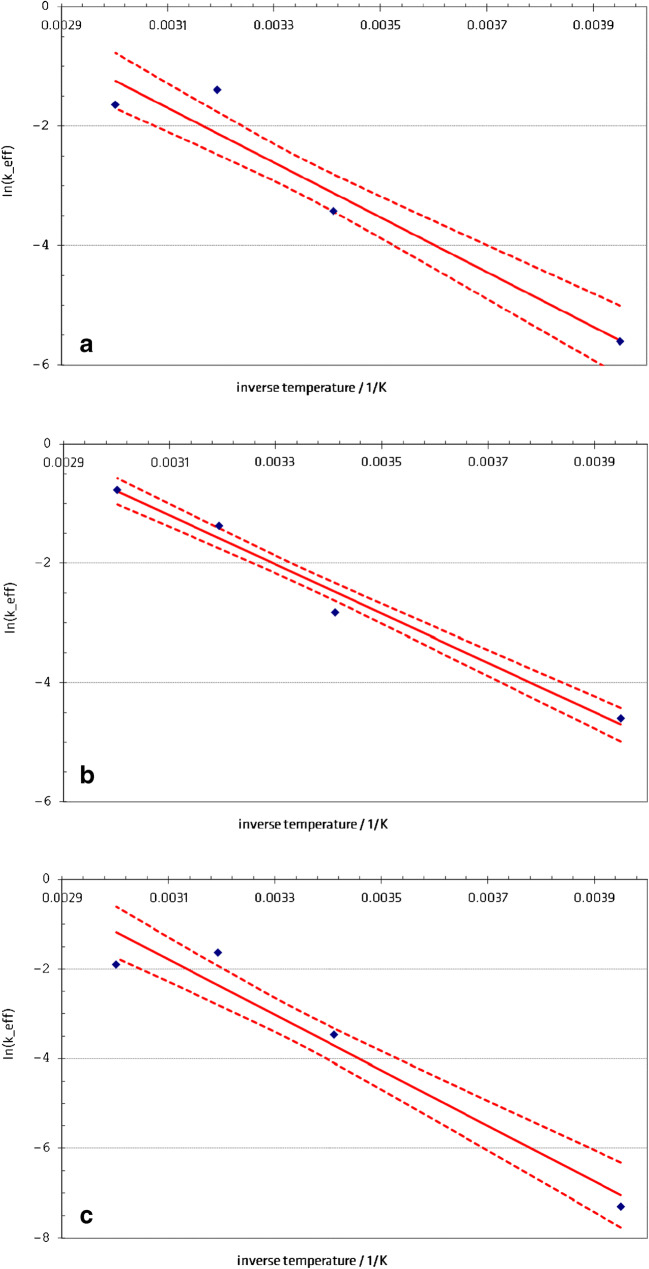
**a**–**c** Effective reaction rate *k*_*eff*_ (T) for acrylamide in cocoa (**a** ERM®-BD513; **b** ERM®-BD514; **c** ERM®-BD515) in dependence on the inverse temperature (semilogarithmic plot). *Solid line* linear regression over the stability data; *dotted lines* upper and lower confidence limits of the regression line

For acrylamide things behaved differently, here a temperature-dependent decrease of the content could already be determined in the short-term stability study. The analytical data obtained for each temperature and time resulted in plots of the logarithms of the reaction rate ln(*k*_eff_) over the inverse temperature for all three cocoa materials (Fig. 1a–c). All temperature dependencies can merely be approximated by a straight line. The corresponding confidence intervals for the lines are also given in the figure/s. The estimated activation energies *∆E* for acrylamide can be calculated to 34 kJ mol^−1^ (ERM®-BD513), 51 kJ mol^−1^ (ERM®-BD514) and 41 kJ mol^−1^ (ERM®-BD515). Since the estimation of *∆E* is based on the data obtained from short-term stability its uncertainty is relatively high. This could be a reason for the high deviations of the estimated values which theoretically should be equal. By using this data and the assumed model, an estimate can be obtained when degradation will presumably force the acrylamide content to fall short of the lower expanded uncertainty limit of the acrylamide content in the three cocoa materials. In the sense of a worst-case estimation, these calculations are carried out for the effective reaction rates at the upper confidence limit of the lines as shown in Fig. 1a–c. The respective shelf lives are given in Table 7. As a result, it is indispensable to store the materials at a temperature of − 21 °C and to ship them cooled. Besides the large scattering of the interlaboratory test results, the results of the stability test were a further reason to state the acrylamide contents only for information and not to certify them. The first estimation of stability will continuously be updated by further measurements of units stored at − 21 °C over the period of availability of the material (post-certification monitoring).

**Table 7:** Estimation of shelf life for acrylamide from short-term stability study

Temperature (°C)	Expiry (month)		
	BD513	BD514	BD515
− 21	78.8	35.0	223
20	8.7	4.3	11.1
40	3.1	1.7	2.7
60	1.1	0.7	0.7

### Characterisation and value assignment

**Fig. 2:**
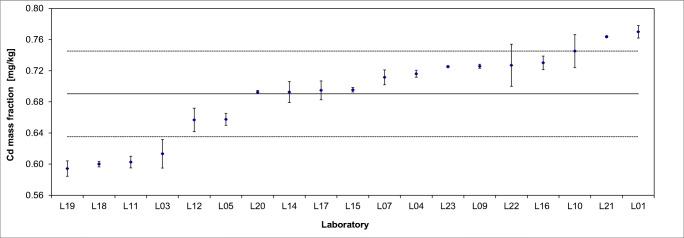
Results of interlaboratory comparison for Cd in ERM®-BD515

**Fig. 3:**
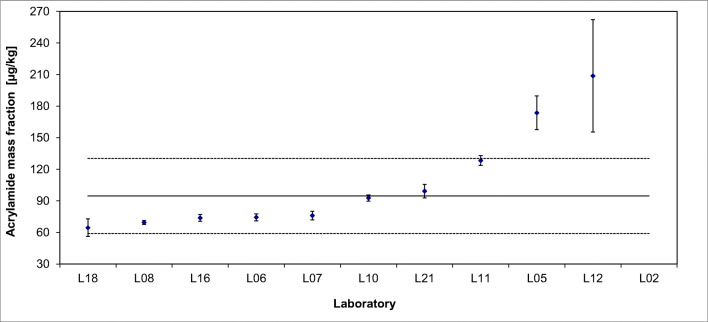
Results of interlaboratory comparison for acrylamide in ERM®-BD515 (Laboratories L12 and L02 (749 μg kg^−1^) were removed as technical outliers)

The results of the interlaboratory comparison of ERM®-BD515 are presented in Fig. 2 for cadmium and in Fig. 3 for acrylamide exemplarily. All data were thoroughly inspected for technical outliers and subsequently tested for statistical outliers (Grubbs, Dixon, Nalimov) using the software program SoftCRM 1.2.2. [20]. No outlying values were detected for Cd but in all three batches acrylamide results of two laboratories were significantly higher and or more widely dispersed than the results of the other participants. Especially Laboratory L02 reported much higher acrylamide contents. It was noticeable that this laboratory weighed in only 0.2 g of sample in contrast to almost all others, whose sample intakes were between 1 and 2 g. A possible calculation error could not be excluded. The results of Laboratory L12 also were higher than those of the other participants and had a wide spread within laboratory, i.e. the method seemed to be not fully under control.

The combined uncertainties were calculated using Eq. 2, considering contributions from the spread resulting from the certification interlaboratory comparison (*u*_ilc_), from possible inhomogeneity (*u*_bb_) and uncertainty terms for purity of calibrant (*u*_pur_) and recovery (*u*_rec_) calculated from the results the participants obtained from their determinations of the control solutions, see Tables 8 and 9. The purity uncertainty contribution *u*_pur_ is the uncertainty of a typical calibration solution, in this case the calibration solution used in our own laboratory (Merck Certipur, Merck for Cd and certified acrylamide standard Ultra Scientific). The recovery uncertainty contribution *u*_rec_ is estimated from the recovery rates obtained in our own laboratory.

**Table 8:** uncertainty contributions and certified values for Cd (all data in mg kg^−1^)

ERM®	Mean*	*u*_ilc_	*u*_bb_	*u*_lts_	*u*_pur_	*u*_rec_	U* (k = 2)
BD513	0.181	0.0043	0.0	0	0.00092	0	0.009
BD514	0.541	0.0106	0.0029	0	0.00275	0	0.024
BD515	0.690	0.0127	0.0040	0	0.00350	0	0.029

*Given as certified values in the certificate of analysis

**Table 9:** uncertainty contributions and indicative values for acrylamide (all data in mg kg^−1^)

ERM®	Mean*	*u*_ilc_	*u*_lts_	*u*_bb_	*u*_pur_	*u*_rec_	U* (k = 2)
BD513	0.051	0.00892	0	0.0	0.00030	0.00073	0.018
BD514	0.101	0.01222	0	0.0023	0.00058	0.00144	0.026
BD515	0.095	0.01187	0	0.0	0.00055	0.00135	0.025

*Given as indicative values in the certificate of analysis

2 $$ {u}_{combined}=\sqrt{u_{ilc}^2+{u}_{bb}^2+{u}_{pur}^2+{u}_{rec}^2} $$

- with

$$ u\mathrm{ilc}=\sqrt{\frac{S_{\mathrm{M}}^2}{n}} $$
uncertainty contribution resulting from interlaboratory comparison
*s*_M_: standard deviation of the laboratories means of accepted data sets
*n*: number of data sets used for calculating the mean of means of each analyte

The certified mass fractions of Cd as well as the informative mass fractions of acrylamide were calculated as arithmetic mean of the accepted data sets (Tables 8 and 9). Different calibrants of known purity and specified traceability of their assigned values were used for calibration and all relevant input parameters were calibrated (see Tables 3 and 4). The individual results are therefore traceable to the SI, as it is also confirmed by the agreement among the technically accepted data sets. As the assigned values are combinations of agreeing results individually traceable to the SI, the assigned quantity values themselves are traceable to the SI as well.

## Conclusions

ERM®-BD513, ERM®-BD514 and ERM®-BD515 represent the first CRMs developed for the determination of Cd in cocoa and considering the maximum content of 0.6 mg kg^−1^ cadmium in cocoa powder defined by the Commission Regulation (EU) No. 488/2014. Additionally, informative values for the acrylamide content are given in all three cocoa materials. The production of the CRMs and certification of the cadmium mass fractions in cocoa matrix was done in compliance with the internationally accepted procedures laid down in ISO Guide 35 [12]. Analyses for homogeneity and stability studies were carried out for Cd using ICP-MS and for acrylamide using LC-MS/MS after appropriate sample decomposition. The certified mass fractions and expanded uncertainties (*k* = 2) of the reference materials were (0.181 ± 0.009) mg kg^−1^ Cd (ERM®-BD513), (0.541 ± 0.024) mg kg^−1^ Cd (ERM®-BD514) and (0.690 ± 0.029) mg kg^−1^ Cd (ERM®-BD514) and are traceable to the SI. The obtained mass fractions for cadmium are based on the results of a certification interlaboratory comparison with experienced laboratories. Owing to the limited number of matrix CRMs available for analysis of cadmium in similar matrices, the CRMs are intended for use in the development and validation of new analytical methods and represent important quality control tools for laboratories to implement and safeguard reliable measurements of cadmium in relevant food matrices in the content area near the defined limit.

## Data Availability

All data and material is available.
