# Correction to: Development of certified reference materials for the determination of cadmium and acrylamide in cocoa

**DOI:** 10.1007/s00216-021-03428-y

**Published:** 2021-06-02

**Authors:** Sebastian Recknagel, Matthias Koch, Robert Köppen, Sabine Buttler, Sibylle Penk, Tatjana Mauch, Thomas Sommerfeld, Angelika Witt

**Affiliations:** Bundesanstalt für Materialforschung und -prüfung (BAM), Richard-Willstätter-Straße 11, 12489 Berlin, Germany

**Correction to: Analytical and Bioanalytical Chemistry (2020) 412:4659–4668**

10.1007/s00216-020-02719-0

The article Development of certified reference materials for the determination of cadmium and acrylamide in cocoa, written by Sebastian Recknagel, Matthias Koch, Robert Köppen, Sabine Buttler, Sibylle Penk, Tatjana Mauch, Thomas Sommerfeld and Angelika Witt, was originally published Online First without Open Access. After publication in volume 412, issue 19, page 4659–4668 the author decided to opt for Open Choice and to make the article an Open Access publication. Therefore, the copyright of the article has been changed to ©The Author(s) 2021 and the article is forthwith distributed under the terms of the Creative Commons Attribution 4.0 International License, which permits use, sharing, adaptation, distribution and reproduction in any medium or format, as long as you give appropriate credit to the original author(s) and the source, provide a link to the Creative Commons licence, and indicate if changes were made. The images or other third party material in this article are included in the article’s Creative Commons licence, unless indicated otherwise in a credit line to the material. If material is not included in the article’s Creative Commons licence and your intended use is not permitted by statutory regulation or exceeds the permitted use, you will need to obtain permission directly from the copyright holder. To view a copy of this licence, visit http://creativecommons.org/licenses/by/4.0.

